# Chemical and Physical Environmental Conditions Underneath Mat- and Canopy-Forming Macroalgae, and Their Effects on Understorey Corals

**DOI:** 10.1371/journal.pone.0012685

**Published:** 2010-09-13

**Authors:** Claudine Hauri, Katharina E. Fabricius, Britta Schaffelke, Craig Humphrey

**Affiliations:** 1 Environmental Physics, Institute of Biogeochemistry and Pollutant Dynamics, ETH Zurich, Zurich, Switzerland; 2 Australian Institute of Marine Science, Townsville, Queensland, Australia; University of Hull, United Kingdom

## Abstract

Disturbed coral reefs are often dominated by dense mat- or canopy-forming assemblages of macroalgae. This study investigated how such dense macroalgal assemblages change the chemical and physical microenvironment for understorey corals, and how the altered environmental conditions affect the physiological performance of corals. Field measurements were conducted on macroalgal-dominated inshore reefs in the Great Barrier Reef in quadrats with macroalgal biomass ranging from 235 to 1029 g DW m^−2^ dry weight. Underneath mat-forming assemblages, the mean concentration of dissolved oxygen was reduced by 26% and irradiance by 96% compared with conditions above the mat, while concentrations of dissolved organic carbon and soluble reactive phosphorous increased by 26% and 267%, respectively. The difference was significant but less pronounced under canopy-forming assemblages. Dissolved oxygen declined and dissolved inorganic carbon and alkalinity increased with increasing algal biomass underneath mat-forming but not under canopy-forming assemblages. The responses of corals to conditions similar to those found underneath algal assemblages were investigated in an aquarium experiment. Coral nubbins of the species *Acropora millepora* showed reduced photosynthetic yields and increased RNA/DNA ratios when exposed to conditions simulating those underneath assemblages (pre-incubating seawater with macroalgae, and shading). The magnitude of these stress responses increased with increasing proportion of pre-incubated algal water. Our study shows that mat-forming and, to a lesser extent, canopy-forming macroalgal assemblages alter the physical and chemical microenvironment sufficiently to directly and detrimentally affect the metabolism of corals, potentially impeding reef recovery from algal to coral-dominated states after disturbance. Macroalgal dominance on coral reefs therefore simultaneously represents a consequence and cause of coral reef degradation.

## Introduction

Disturbance of coral reefs by nutrient enrichment, sedimentation, overfishing and a warming climate have become more frequent and more severe over the past decades. These disturbances can stress or kill corals and lead to substratum becoming available for colonization. After initial colonization by microalgae, fast growing macroalgal assemblages often dominate the newly available substratum during later successional stages [1].

**Figure 1:**
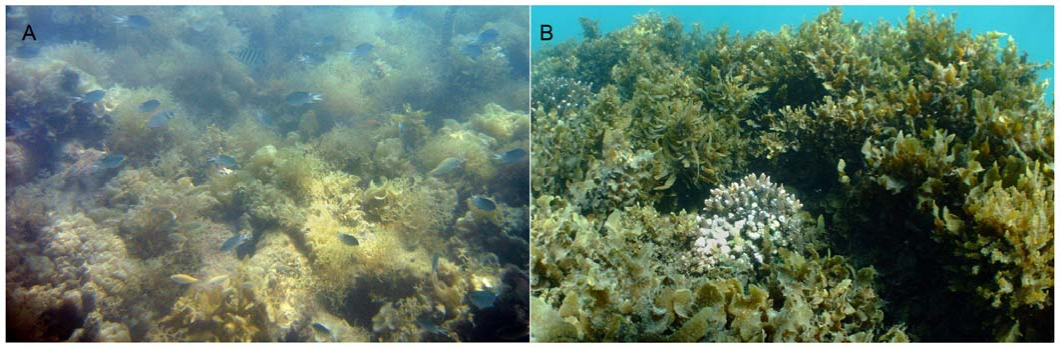
Macroalgae assemblages at Long Island and Dunk Island. (A) Mat-forming macroalgal assemblage dominated by *Hydroclathrus clathratus*, Long Island. (B) Canopy-forming macroalgal assemblage, dominated by *Sargassum spp*., Dunk Island.

On the Great Barrier Reef (GBR), ephemeral (seasonal or episodic) macroalgal blooms can occur even in coral-dominated areas [2]. These ephemeral blooms can cover large areas of substratum on flats and crests of coastal and inshore fringing reefs, predominantly from early spring to early summer, often blanketing small corals and other sessile coral reef organisms [2]. Many of these ephemeral macroalgae form dense carpet-like mats, 10–50 cm thick, that are only loosely attached to the substratum (e.g., *Hydroclathrus clathratus*; Fig. 1A). A large number of GBR inshore reefs also have assemblages of large canopy-forming and often perennial macroalgae (mainly brown macroalgae, e.g. the genus *Sargassum*), with maximum biomass and height (0.5–1.5 m) in late summer (Fig. 1B). Mixed assemblages consisting of both ephemeral and perennial mat- and canopy-forming taxa are also common.

As macroalgae colonize potential settlement substratum for corals, they are assumed to hamper the recovery of coral assemblages after disturbance [3]. Corals and macroalgae interact and compete in a variety of ways. Previous studies have mainly focused on the response of corals to direct physical contact with macroalgae. Direct overgrowth of living corals by macroalgae can result in reduced coral growth caused by chronic polyp retraction and tissue loss [4], coral disease [5] or even mortality [6]. Competition by allelopathy was confirmed for the red alga *Plocamium hamatum* affecting the soft coral *Sinularia cruciata* [7], and has been implied as the cause of tissue necrosis in corals that were in contact with the filamentous red alga *Anotrichium tenue* [8] or the filamentous cyanobacterium *Lyngbya bouillonii* [9]. Other chemical and biological interactions between corals and algae are likely. For example, Smith et al. [10] observed oxygen depletion and subsequent death of corals that were placed in close proximity to algae in a small-scale laboratory experiment. The authors suggested that the algae released high concentrations of dissolved organic carbon from excess photosynthates, which may have lead to high microbial activity affecting the corals [11]. Macroalgal mats or canopies may alter the physical and chemical microenvironment for the benthic fauna and flora underneath, possibly to their detriment. Steep vertical oxygen profiles have been recorded within macroalgal assemblages, due to high photosynthetic rates in the well-lit upper layers and reduced photosynthesis from self-shading in deeper layers [12]. Algal mats or canopies and seagrass meadows can also reduce water flow by up to 90% [13], potentially leading to reduced gas exchange and an accumulation of nutrients, metabolic waste products and carbon (e.g. leached excess photosynthates and animal waste products).

The objectives of this study were to: i) characterize the physical and chemical properties of the microenvironment underneath macroalgal assemblages in a coral reef environment; ii) evaluate how macroalgal morphology (mats versus canopies) and biomass influences this microenvironment; and iii) investigate maximum quantum yield (photosynthetic activity), RNA/DNA ratio and survival rates as physiological responses of corals exposed to this microenvironment.

## Results

### Field study: Biomass and species composition of macroalgal assemblages

A total of 28 macroalgal taxa were identified. Mat-forming assemblage types (Fig. 1A) were dominated by the ephemeral species *Hydroclathrus clathratus*, intermixed with *Galaxaura obtusata*, *Lobophora variegata*, *Halimeda* sp., *Padina* sp., *Sporochnus* sp., *Botryocladia leptopoda*, *Dictyota* sp., *Hormophysa cuneiformis*, *Lobophora variegata*, *Digenea simplex* and small thalli of *Sargassum* spp. and *Cystoseira trinodis*, with an average diversity of 6.8 taxa ±1.6 SD per 0.25 m^2^ quadrat. Canopy-forming assemblage types (Fig. 1B) were dominated by the tall and frondose perennial taxa *Sargassum fissifolium*, *S. polycystum*, *S. oligocystum*, *S. deccurens*, *S. siliquosum*, *Sargassum* spp. and *Hormophysa cuneiformis*, with some *Lobophora variegata*, *Jania adhaerens* and *Padina* spp. as understorey components, with an average diversity of 4.7 taxa ±1.6 SD per 0.25 m^2^ quadrat. The biomass of macroalgae varied widely between quadrats, ranging from 235 g DW m^−2^ to 1029 g DW m^−2^ (sample mean: 576 g DW m^−2^ ±241 SD). Quadrats with canopy-forming assemblages had higher mean biomass (630 g DW m^−2^ ±257 SD, n = 11) than quadrats with mat-forming assemblages (440 g DW m^−2^ ±153 SD, n = 8).

### Physical and chemical parameters underneath and above algal assemblages

**Figure 2:**
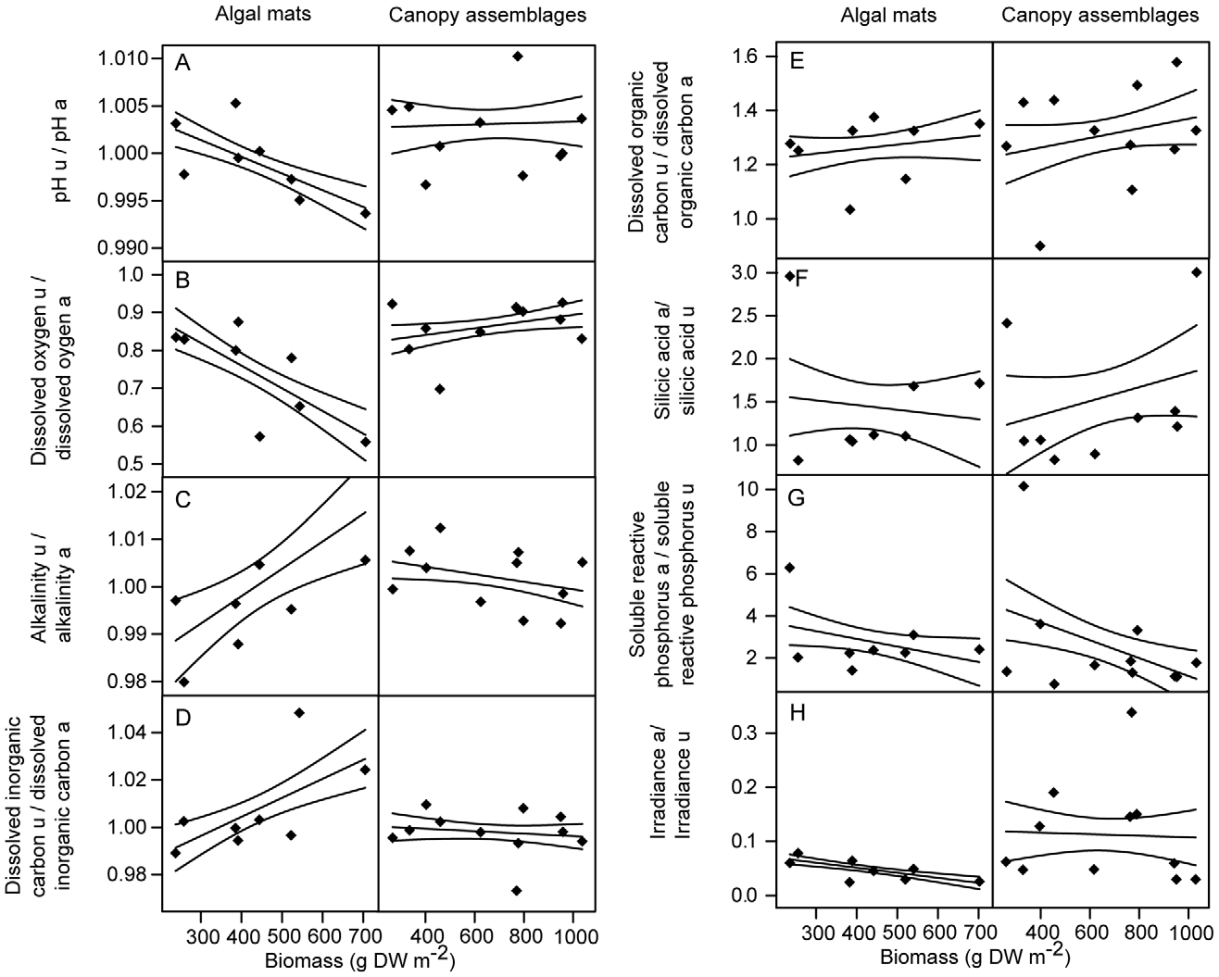
The effects of macroalgal biomass and assemblage type on the physical and chemical conditions underneath and above macroalgal assemblages in the field on inshore coral reefs. Ratio of (A) pH, (B) dissolved oxygen (DO, mg L^−1^), (C) alkalinity (ALK, µEq/kg), (D) dissolved inorganic carbon (DIC, µM), (E) dissolved organic carbon (DOC, mg L^−1^), (F) silicic acid (Si, µM), (G) soluble reactive phosphorus (SRP, µM) and (H) irradiance (µmol photons m^−2^ s^−1^) underneath (u) and above (a) macroalgal assemblages as a function of macroalgal biomass and assemblage type (mat-forming vs canopy-forming assemblages; see also Table 2). Lines are linear model fits and 95% confidence intervals.

Dense mats and canopies of macroalgae created a microenvironment with a relatively small volume of water around the understorey corals. In both macroalgal assemblages types, irradiance and concentrations of dissolved oxygen (DO) and dissolved organic carbon (DOC) were significantly different underneath and above the assemblages, while alkalinity, dissolved inorganic carbon (DIC), pH and silicic acid (Si) were similar (Table 1). The irradiance underneath the macroalgae was reduced to 4–5% of values above the macroalgae. DO concentrations were 26% and 13% lower underneath than above mat-forming and canopy-forming assemblages, respectively. DOC concentrations were between 26% (mat-forming) and 30% (canopy-forming) higher and more variable underneath the assemblages, while SRP was 2.7 times higher underneath than above mat-forming assemblages, with no significant difference in canopy-forming assemblages. Ratios of physical and chemical parameters underneath to above algal assemblages were used to assess the effects of algal biomass and assemblage type. The ratio of DO concentration underneath to above the assemblages showed a strong interaction between type (mats vs canopies) and biomass, with DO ratios strongly decreasing with increasing biomass in algal mats but not in canopies (Table 2, Fig. 2B). The ratios of alkalinity and DIC concentration underneath to above the assemblages showed a weaker, but nonetheless significant, interaction between type and biomass. Both ratios increased with increasing biomass in algal mats, while the ratios remained constant in the canopy assemblages (Table 2, Fig. 2C and D). Irradiance, pH and concentrations of DOC and SRP showed no relationship to biomass or assemblage type (p>0.05;Table 2, Fig. 2).

**Table 1:** Physical and chemical conditions underneath and above macroalgal assemblages in the field on inshore coral reefs.

	Mean above (±SE)	Mean underneath (±SE)	Estimated Difference (u – a; %)	t		P
Mat-forming macroalgal assemblages						
pH	8.20±0.01	8.19±0.01	−0.10		−0.731	0.488
DO	6.12±0.11	4.52±0.30	−26		−5.94	<0.001
ALK	2230±9	2230±8	0		−0.004	0.997
DIC	1797±5	1810±12	0.72		1.05	0.329
DOC	0.69±0.01	0.87±0.02	26		6.46	<0.001
Si	1.23±0.03	1.74±0.28	42		1.8	0.117
SRP	0.06±0.07	0.16±0.03	267		3.08	0.018
Irradiance	225±34	8.96±0.51	−96		−6.4	<0.001
Canopy-forming macroalgal assemblages						
pH	8.17±0.01	8.20±0.01	0.31		−0.732	0.488
DO	6.15±0.13	5.33±0.21	−13		−6.99	<0.001
ALK	2170±14	2174±14	0.21		1.07	0.308
DIC	1756±11	1752±14	−0.19		−0.67	0.52
DOC	0.73±0.01	0.95±0.05	30		5.47	<0.001
Si	3.84±0.66	5.32±1.15	38		1.26	0.238
SRP	0.09±0.02	0.11±0.02	21		1.65	0.13
Irradiance	299±88	16.4±2.01	−95		−3.28	0.008

Physical and chemical conditions underneath and 0.3 m above mat- (N = 8) and canopy-forming (N = 11) macroalgal assemblages on inshore coral reefs of the Great Barrier Reef, and results of paired t-tests. Abbreviations and units: pH, dissolved oxygen (DO, mg L^−1^), alkalinity (ALK, µEq/kg), dissolved inorganic carbon (DIC, µM) dissolved organic carbon (DOC, mg L^−1^), silicic acid (Si, µM), soluble reactive phosphorus (SRP, µM) and Irradiance (µmol photons m^−2^ s^−1^).

**Table 2:** The effects of macroalgal biomass and assemblage type on the physical and chemical conditions in the field on inshore coral reefs.

		df	MS	F	p
pH	Assemblage type	1	<0.0001	4.11	0.0608
	Biomass	1	<0.0001	0.344	0.5663
	Assemblage type * Biomass	1	<0.0001	2.42	0.1410
	Residuals	15	<0.0001		
DO	Assemblage type	1	0.0750	12.3	0.0031
	Biomass	1	0.0010	0.237	0.6338
	Assemblage type * Biomass	1	0.0640	10.6	0.0053
	Residuals	15	0.0060		
ALK	Assemblage type	1	<0.0001	0.170	0.6863
	Biomass	1	<0.0001	0.156	0.6985
	Assemblage type * Biomass	1	0.0006	5.48	0.0334
	Residuals	15	0.0001		
DIC	Assemblage type	1	0.0004	2.35	0.4160
	Biomass	1	0.0001	0.612	0.4462
	Assemblage type * Biomass	1	0.0010	5.85	0.0288
	Residuals	15	0.0025		
DOC	Assemblage type	1	0.0102	0.361	0.5567
	Biomass	1	0.0274	0.972	0.3398
	Assemblage type * Biomass	1	<0.0001	<0.001	0.9793
	Residuals	15	0.0281		
Si	Assemblage type	1	0.0653	0.079	0.7832
	Biomass	1	0.2764	0.332	0.5730
	Assemblage type * Biomass	1	0.2492	0.299	0.5923
	Residuals	15	0.8326		
SRP	Assemblage type	1	0.2341	0.049	0.8280
	Biomass	1	15.2720	3.19	0.0944
	Assemblage type * Biomass	1	0.0470	0.010	0.9224
	Residuals	15	4.7895		
Irradiance	Assemblage type	1	0.0191	3.26	0.0912
	Biomass	1	0.0008	0.129	0.7246
	Assemblage type * Biomass	1	0.0008	0.144	0.7096
	Residuals	15	0.0059		

Results of two way ANOVA comparing the effects of macroalgal assemblage type (mat- or canopy forming) and macroalgal biomass on the ratios (underneath vs above) of pH, dissolved oxygen (DO, %) alkalinity (ALK, µEq/kg), dissolved inorganic carbon (DIC, µM), dissolved organic carbon (DOC, mg L^−1^), silicic acid (Si, µM), soluble reactive phosphorus (SRP, µM) and irradiance (µmol photons m^−2^ s^−1^) (see also Fig. 2).

### Aquarium experiment: Characteristics of the simulated microenvironment

**Figure 3:**
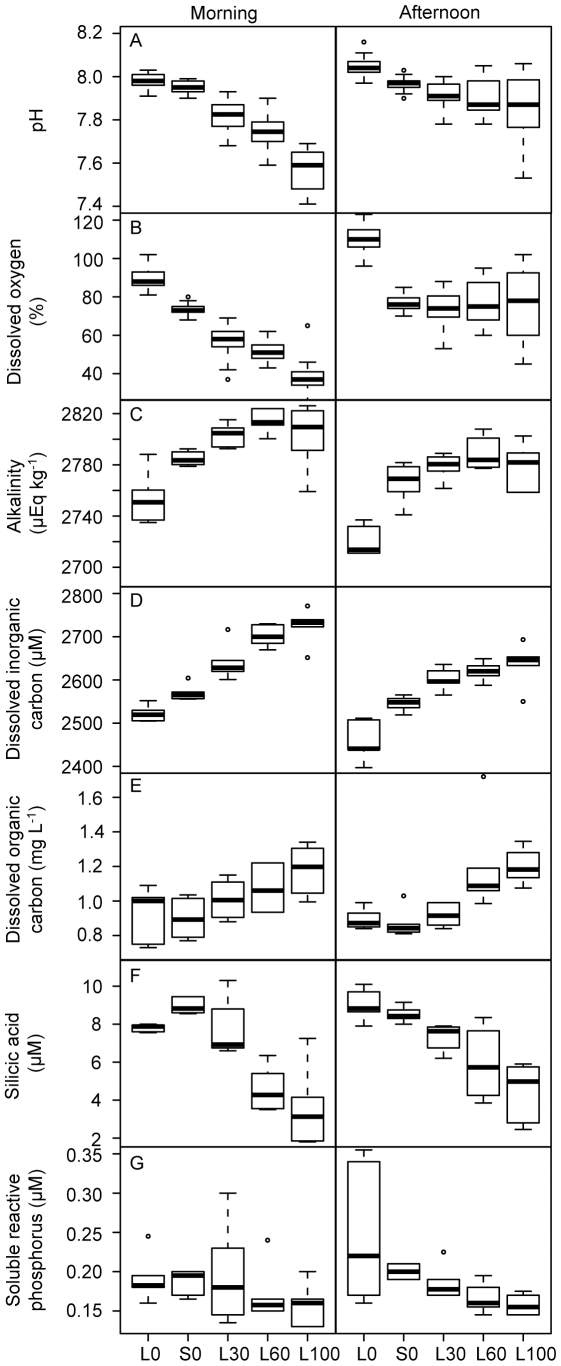
The effects of macroalgae on water chemistry in the laboratory experiment. (A) pH, (B) dissolved oxygen saturation (DO, %), (C) alkalinity (ALK, µEq/kg), (D) dissolved inorganic carbon (DIC, µM), (E) dissolved organic carbon (DOC, mg L^−1^), (F) silicic acid (Si, µM) and (G) soluble reactive phosphorus (SRP, µM) in different experimental treatments, measured in the morning and in the afternoon of days 2 and 6 (see Table 3 and 4 for details). Treatments are light (L) and shading (S) (white and grey boxes, respectively), at increasing concentrations of algal incubated water (0%, 30%, 60% and 100% addition).

The water pre-incubated with macroalgae had elevated alkalinity, DIC and DOC concentrations and reduced pH, DO saturation and Si concentrations compared with control water. A comparison of the shaded and illuminated controls (0% algal water) showed significant interactions between irradiance and time of sampling in pH, DO and Si values, with higher pH in the illuminated controls in the afternoon and higher DO in the illuminated controls at both times (Table 3, Fig. 3). A comparison of the shaded treatments showed significant interactions between concentration and time of sampling in pH and DO. With increasing algal water concentration pH decreased, with greater differences in the morning than in the afternoon (Table 4, Fig. 3). DO saturation was reduced depending on algal water concentration in the morning, but not in the afternoon. Concentration of DOC increased slightly while Si and SRP decreased with increasing algal water concentration at both sampling times.

**Table 3:** The effects of time of sampling (morning vs. afternoon) and treatment factors (light vs shade) on water chemistry in the laboratory experiment.

		pH		DO			ALK		DIC		DOC		Si		SRP	
	df	F	p	F	p	df	F	p	F	p	F	p	F	p	F	p
Irradiance	1	66.2	<0.0001	703	<0.0001	1	29.7	<0.0001	38.4	<0.0001	0.418	0.5251	2.81	0.1092	0.056	0.8151
Time	1	40.5	<0.0001	162	<0.0001	1	14.8	0.0010	16.5	0.0006	0.639	0.4336	0.477	0.4979	2.41	0.1360
Irradiance:Time	1	19.2	<0.0001	88.8	<0.0001	1	2.44	0.1337	3.49	0.0765	0.009	0.9252	11.2	0.0033	0.017	0.8966
Residuals	109					20										

pH, dissolved oxygen saturation (DO, %), alkalinity (ALK, µEq/kg), concentrations of dissolved inorganic carbon (DIC, µM), dissolved organic carbon (DOC, mg L^−1^) silicic acid (Si, µM) and soluble reactive phosphorus (SRP, µM) in the treatment units. Results of linear mixed-effects models comparing controls only (no algal water added; treatment  =  light versus shade).

**Table 4:** The effects of time of sampling (morning vs. afternoon) and treatment factors (algal water concentrations on water chemistry in the laboratory experiment.

		pH		DO			ALK		DIC		DOC		Si		SRP	
	df	F	p	F	p	df	F	p	F	p	F	p	F	p	F	p
Concentration	1	288	<0.0001	112	<0.0001	1	0.534	0.4686	88.5	<0.0001	34.5	<0.0001	85.1	<0.0001	6.99	0.0113
Time	1	156	<0.0001	275	<0.0001	1	15.3	0.0003	37.0	<0.0001	0.0084	0.9273	0.469	0.4970	0.265	0.6096
Concentration:Time	1	74.3	<0.0001	103	<0.0001	1	1.43	0.2384	7.24	0.0101	0.8468	0.3625	3.31	0.0758	0.825	0.3687
Residuals	223					44										

pH, dissolved oxygen saturation (DO, %), alkalinity (ALK, µEq/kg), concentrations of dissolved inorganic carbon (DIC, µM), dissolved organic carbon (DOC, mg L^−1^) silicic acid (Si, µM) and soluble reactive phosphorus (SRP, µM) in the treatment units. Results of linear mixed-effects models comparing shaded treatments with 0%, 30%, 60% or 100% algal water concentration.

### Coral response

On day 7, two corals in one of the beakers containing 100% algal water had died (complete tissue loss) and were removed. On day 10, the third coral from the same replicate beaker, and one coral from another beaker of the same treatment, had lost tissue at the base. No visible effects were observed in any of the other treatments.

**Figure 4:**
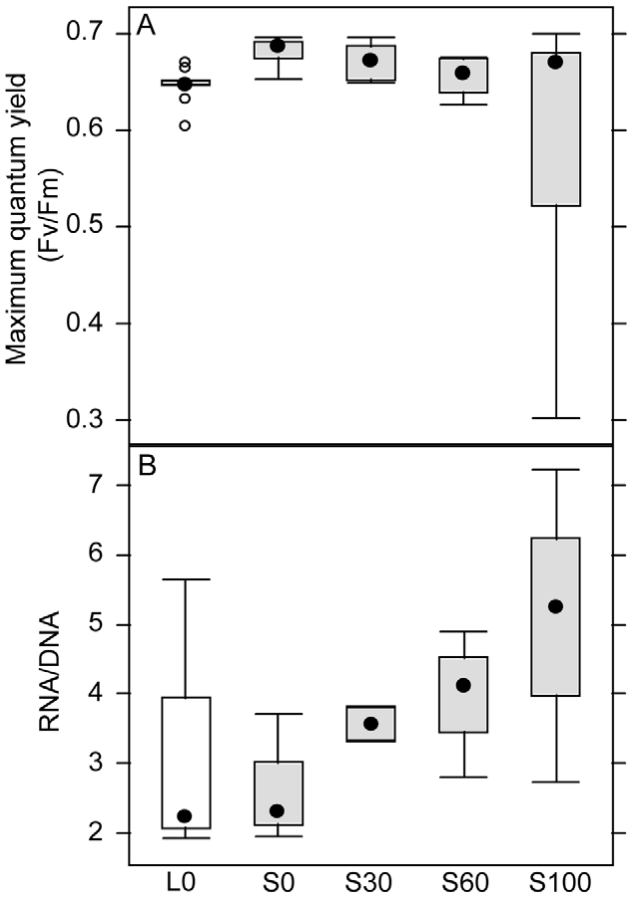
The effects of macroalgae on maximum quantum yield and RNA/DNA ratio of corals in the laboratory experiment. (A) Maximum quantum yield (Fv/Fm) and (B) RNA/DNA ratio in nubbins of the coral *Acropora millepora* after 10 days of exposure to microenvironments simulating conditions underneath macroalgal assemblages. Data are untransformed means ± SE (n = 9; and n = 6 in the S100 treatment).

Maximum quantum yields of the surviving corals were measured on days 0, 3, 7 and 10 (data from day 10 presented in Table 5 and Fig. 4A). Corals in the illuminated control beaker had a significantly lower maximum quantum yield than the corals in the shaded control beaker. In the shaded treatments the maximum quantum yield decreased linearly with increasing algal water concentration.

**Table 5:** Treatment effects on maximum quantum yields and RNA/DNA ratios in corals in the aquarium experiment.

	Maximum quantum yield				RNA/DNA			
	df	MS	F	p	df	MS	F	p
Irradiance	1	0.0036	21.981	0.0002	1	0.0276	0.1475	0.7205
Residuals	16	0.0002			4	0.1874		
Concentration	1	0.0337	7.652	0.0088	1	0.5254	5.4275	0.0448
Residuals	37	0.0044			9	0.0968		

Results of linear models testing for the effects of light (illuminated versus shaded controls) and concentrations (4 concentrations of algae-incubated water in shaded conditions) on the maximum quantum yield (arc-sin square-root transformed) and RNA/DNA ratios (square-root transformed) in coral nubbins at the end of the experiment.

Changes in the RNA/DNA ratios in coral tissues were also related to the concentration of added algal water (Table 5). RNA/DNA ratios increased linearly with increasing algal water concentration (Fig. 4B), whereas RNA/DNA ratios did not differ between corals in shaded or illuminated control treatments.

## Discussion

Our study showed that dense assemblages of macroalgae found on inshore reefs in the GBR significantly alter the physical and chemical microenvironment underneath their mats or canopies. The results from the laboratory experiment agreed well with the findings from the field study. In the field, algal mats and their biomass had a positive effect on alkalinity and DIC, while decreasing light and DO. The same was found in the laboratory experiment, which produced a more pronounced effect on the chemical variables, probably as a result of a slightly lower rate of water exchange. These results provide a better understanding of the processes leading to persistent macroalgal dominance on coral reefs after coral mortality events.

Most of the recorded conditions underneath macroalgal assemblages are known to reduce coral growth through a number of pathways. For example, severe shading, as recorded under the algal assemblages, leads to decreased rates of photosynthesis [14] and calcification [15]. The minimum downward irradiance required for coral reef development at inshore reefs of the Great Barrier Reef is 6–8% of surface irradiance [16] and irradiance below this level can cause severe photo-physiological stress in some corals [17]. In contrast, a moderate level of shading, such as a light reduction by 30–40% reported under canopy-forming *Sargassum hystrix* did not affect short-term growth rates of *Porites sp*. [4]. A change in the spectral composition of light can alter maximum quantum yields in some coral species [18], but to a lesser extent than the 95% reduction in light availability recorded here.

Macroalgae are known to reduce water flow within their assemblages [13], which directly affects the primary productivity and metabolic rates of reef communities [19]. Reduced water exchange with the surrounding water also leads to accumulation of metabolic products of both macroalgae and understorey corals. Using DO concentration as a measure of water exchange rates, our data suggest that mat-forming algae restrict water exchange more than canopy-forming algae, and that the rate of water exchange declines with increasing biomass of these algal mats. Flow measurements underneath *Caulerpa* and seagrass canopies showed that the degree of flow restriction was dependent on the physical structure of the organisms [20]. Low DO concentrations underneath the algae, especially at night, are likely to limit coral respiration [21] and to reduce the metabolism of zooxanthellae [22]. This may be exacerbated at low flow, which increases the diffusive boundary layer and impedes oxygen diffusion.

Alkalinity and DIC are related to calcification and dissolution of CaCO_3_ and organic matter production and remineralization. As dissolution of CaCO_3_ takes place, alkalinity and DIC strongly increase. Organic matter production also has a positive, but very weak effect on alkalinity, while it strongly decreases DIC [23]. Reduced pH and resulting low carbonate ion (CO_3_^2−^) concentrations negatively affect coral calcification, as shown in the context of ocean acidification [15] and can lead to dissolution of the CaCO_3_ coral skeleton and reef substratum. Our calculated values for pH from the field study suggest that corals underneath thick algal mats are exposed to lower pH than found in ambient water on inshore coral reefs. These findings are further supported by two semi-continuous 24 h measurements underneath and above algal mats with a pair of DataSonde® 4 (Aqualab Scientific) sensors. Although these field measurements were insufficiently replicated for precise estimates, both runs suggested pH to be up to ∼0.15 units lower underneath than above the two mats, and ∼0.1 units lower at night than during the day (data not shown). The aquarium experiment showed a significant reduction in pH in treatments exposed to macroalgae, especially in the early morning measurements. Field and aquarium results also showed increased levels of alkalinity and DIC, associated with macroalgae. Our data suggest that corals growing amongst macroalgal assemblages experience low pH, especially at night, potentially inhibiting their calcification and enhancing dissolution of their CaCO_3_ skeleton. Elevated SRP, as recorded underneath the macroalgal assemblages, can also reduce coral growth, while enhancing net photosynthesis and nutrient content of macroalgae [24].

High levels of DOC, possibly from the release of macroalgal or coral photosynthates [25] have been shown to enhance microbial activity on coral surfaces, leading to oxygen depletion [10], microbial digestion of coral polyps and accumulation of secondary metabolites [26]. Elevated levels of DOC have also been associated with coral diseases and the progressive loss of coral tissue [11]. It is likely that allelopathic substances and other secondary metabolites excreted by macroalgae and sessile understorey organisms further reduced the suitability of the chemical microenvironment for coral growth; however, these compounds were not analyzed here.

In this study, negative effects on coral health were documented by measuring maximum quantum yields and RNA/DNA ratios. Low maximum quantum yields of the endosymbiotic dinoflagellates are commonly interpreted as signs of photophysiological stress. Stress from high light is known to affect maximum quantum yields [27], and indeed the maximum quantum yields in the illuminated control dropped during the first three days due to light stress, and recovered only partially after the irradiance was reduced. However, in shaded conditions, maximum quantum yields also decreased linearly with increasing concentrations of algae-incubated water indicating that the altered chemical environment also affected the photo-physiology of the exposed corals.

The increasing RNA/DNA ratio at increasing concentrations of macroalgal-incubated water also indicated that the metabolism of the corals was affected by the presence of macroalgae. RNA concentrations vary in relation to protein synthesis, while DNA concentrations remain constant. Transplant studies on corals show that the RNA/DNA ratios decrease with decreasing light, suggesting that lower irradiance leads to reduced growth [28]. However, the positive correlation between macroalgal water concentration and RNA/DNA ratios in the laboratory experiment indicates the synthesis of new proteins. Since yields decreased concomitantly with increasing algal water concentration, the increasing RNA/DNA ratios possibly reflected the synthesis of stress proteins in response to changes in the microenvironment by the algae. Corals are known to synthesize a range of proteins in response to heat stress, exposure to copper ions and starvation [29]–[31]. Increased microbial growth may have also contributed to the observed increase in the RNA/DNA ratio; however, their contribution is likely to have been small given the much larger biomass of coral tissue and zooxanthellae.

Our study suggests that the dominance of macroalgae on coral reefs may simultaneously represent a consequence and cause of coral reef degradation [32]. In particular ephemeral macroalgal mats, which severely restrict water exchange, may cause significant physiological stress to understorey corals. In contrast, assemblages of perennial canopy-forming macroalgae, while also decreasing light and increasing DOC for understory corals, seem to allow slightly greater water exchange, and hence cause less stress in corals. Both our laboratory and field study showed that not only algal cover, but also the algal biomass per unit area (i.e., the thickness of algal mats) is an important factor in determining how much the understory chemical microenvironment is altered. As competitive interactions between corals and macroalgae become more frequent and of longer duration ([33]; but see [34]), it is necessary to understand the underlying mechanisms that lead to shifts in competitive ability between these groups. We propose here that macroalgae do not only benefit from and/or tolerate conditions that cause stress in corals, but that algal mats have direct negative effects on the physiology of corals, which may explain the persistence of algal-dominated community states on some reefs.

## Materials and Methods

This study was approved as part of ongoing research of the Australian Institute of Marine Science.

### Field study

We determined physical and chemical parameters underneath and above dense macroalgal assemblages with different taxonomic composition, morphologies and biomass. Field data were collected on fringing coral reefs around four inshore islands of the Great Barrier Reef (GBR), 4 to 15 km off the Australian coast, in January and February 2007. The sampling locations were Lindeman, Repulse and Long Islands (Whitsunday Islands, 20° 00′ S, 148° 45′ E) and Dunk Island (17° 56′ S, 146° 08′ E). Due to the heterogeneous nature of macroalgal cover, sample locations were selected by visual inspection, typically in 2 to 8 m water depth. After visually identifying assemblage type, 19 sub-sections for analysis were defined by the haphazard placement of a 50×50 cm stainless steel quadrat on top of a mat.

In each quadrat we determined irradiance, DO, salinity and temperature in three pairs of measurements both underneath and 0.3 m above the macroalgae. Irradiance was measured as photosynthetically active radiation (PAR) using the small light sensor (1 mm diameter) of a Diving PAM (Heinz Walz GmbH) strapped onto a ruler and held horizontally, which was calibrated against a LI-192 light sensor (LI-COR, Nebraska, USA). DO concentration, salinity and temperature were measured at ambient flow with the slim sensor of a hand-held instrument (YSI Model 55 Handheld Dissolved Oxygen System) customized for underwater use. To determine concentrations of SRP, Si and DOC, duplicate water samples were collected by filling 60 mL acid-washed plastic syringes with seawater from underneath and above the macroalgae. To determine DIC concentrations and alkalinity (to calculate pH, see below), water was collected directly into duplicate screw-top plastic test tubes, avoiding the formation of air bubbles. Sensors, syringes and tubes were carefully inserted underneath the assemblages to minimize mixing. After the water sampling, all macroalgae within the quadrats were collected.

Immediately after the collection, samples for SRP, silicic acid and DOC were filtered (0.45 µm Minisart®, Sartorius) into duplicate 10 mL plastic screw-top test tubes. DOC samples were fixed with 100 µL concentrated HCl after filtration. DIC and alkalinity samples remained unfiltered and were analyzed within 14 days. Samples were stored at room temperature (Si), 4°C (DOC, alkalinity, DIC) or −20°C (SRP) until analysis (see below).

The collected macroalgae were washed in seawater to remove adhering sediment, sorted and identified to the highest possible taxonomic level, limited by field conditions (usually genus or species, [35], [36]). Assemblages of each quadrat were categorized as mat-forming or canopy-forming and the dry weight (DW) was determined (drying at 60°C for 4 days of all taxa belonging to either morphological group).

### Aquarium experiment

In a 10-day laboratory study corals were exposed to treatments simulating the conditions recorded underneath macroalgae assemblages in the field study. The chemical microenvironment was simulated by mixing water from a 100 L pre-incubation container containing *Sargassum baccularia* and *Hormophysa triquetra* (145 and 67 g dry weight, respectively) and a second 100 L pre-incubation container without macroalgae. Seawater filtered through a series of 10, 5 and 0.1 µm cartridge filters and an activated carbon filter (Watermart) was supplied at 150 mL min^−1^ to the two 100 L pre-incubation containers. Concentrations of DOC, SPR and Si in the incoming seawater were higher than those recorded around the inshore islands, reflecting the coastal origin of the incoming seawater supply during the wet season. Both containers received 400 µmol photons m^−2^ s^−1^ (fluorescent aquarium lamps, CA® PL-L, 96 W, 10 000 k, photo period: 12 h light, 12 h dark), and water circulation was provided by submersible pumps. The light environment for the experimental treatments was simulated using two densities of shade cloth, resulting in 300 µmol photons m^−2^ s^−1^ for illuminated treatments (425 µmol photons m^−2^ s^−1^ for the first three days) and 4 µmol photons m^−2^ s^−1^ for shaded treatments (fluorescent lamps as above).

Five treatments were established, each represented by three replicate 500 mL glass beakers, which each contained three replicate coral nubbins. Coral nubbins were produced by attaching short branches (3±1 cm in length) of the coral species *Acropora millepora* to 1 cm^2^ plastic bases with non-toxic modeling clay (Newbound), and kept in running seawater for two weeks to recover. The five treatments included two controls (0% addition of algal-incubated water, with and without shading), and 36±2%, 67±4% and 100% of algal-incubated water with shading. Concentrations were obtained by mixing pre-incubated water with and without macroalgae using peristaltic pumps (Masterflex, L/S Digital Standard Drive). Continuous inflow created gentle water flow and exchanged the water in each beaker 50±2 times daily. The water temperature was 25.2±0.5°C in all treatments throughout the experiment.

Every day, pH, DO (saturation) and temperature were monitored with handheld instruments at the end of the 12 h dark period (‘morning’) and after 8 h of light (‘afternoon’). Water samples for analysis of alkalinity and concentrations of DOC, SRP and silicic acid were collected as described above, on days 2 and 6 in the morning and in the afternoon. The pre-incubation containers were cleaned daily to reduce fouling and to remove any decaying biomass from the algal tank. The condition of the corals was monitored by visual assessments and by measuring their maximum quantum yields and RNA/DNA ratios. The maximum quantum yield of the corals was measured on days 0, 3, 7 and 10 with a pulse-amplitude-modulated fluorometer (DIVING-PAM, Walz, Germany; [37]). The maximum quantum yield of dark-adapted corals was determined at the end of the dark period, with five measurements taken at a distance of 3 mm from each nubbin. At the end of the experiment coral nubbins were snap frozen in liquid nitrogen and stored at −80°C for later analysis of RNA/DNA ratios.

### Analysis of RNA/DNA ratios in corals

All coral nubbins (∼2 cm long) were crushed in liquid nitrogen ensuring that the samples remained frozen. The powdered coral (∼2 g) was added to 10 mL of extraction buffer (Tris-EDTA (TE) buffer [10 mM Tris-HCl, 1 mM EDTA, pH 7.5] with 1% sarcosyl). A blank control was added that contained only the extraction buffer and was treated in the same manner as the samples. The samples and blanks were sonicated in an ice bath for 30 s and then centrifuged for 3 min at 1200 × *g* to remove skeletal material. 15 µL of the supernatant was placed in a deep well plate (Megatiter Plate 2.2 mL Deep Well, Polypropylene, sterile) with 1485 µL of TE buffer and mixed thoroughly. Methods for the determination of RNA and DNA were modified from Kyle et al. [38]. Three black 96-well microplates were prepared by adding to each plate 75 µL of nucleic acid standards (0–1.5 µg mL^−1^ for DNA and RNA), and control homogenate or sample. Plate 1 had 15 µL of TE buffer and 75 µL of RiboGreen® solution added. Plate 2 had 7.5 µL of TE buffer and 7.5 µL of RNase, which was allowed to incubate at room temperature for 40 min before adding 75 µL of RiboGreen®. Plate 3 had 7.5 µL of RNase and 7.5 µL of DNase added and allowed to incubate at room temperature for 60 min before adding 75 µL of RiboGreen®. Each microplate was placed into a BioTek Synergy HT microplate reader at 25°C and gently shaken before being read at 485 nm (excitation) and 528 nm (emission). Fluorescence due to RNA was calculated by subtracting the fluorescence of Plate 2 from that of Plate 1 and fluorescence due to DNA was calculated by subtracting the fluorescence of Plate 3 from that of Plate 2. Concentrations of RNA and DNA were calculated using nucleic acid standard curves from each plate.

### Analysis of water samples

SRP and Si concentrations in the seawater were determined by standard wet chemical methods [39] implemented on a segmented flow analyser (Bran and Luebbe AA3). Alkalinity was determined by automatic titration (TT-Processeur-2, Tacussel Electronique; [40]). Dissolved inorganic carbon DIC (from field study) and DOC concentrations were determined on a Shimadzu analyser (TOC 5000A). DIC samples were injected into a 20% phosphoric acid solution to convert DIC to CO_2_, which was delivered by a carrier gas stream to the non-dispersive infrared detector. Calibration standards were prepared from a mixture of sodium bicarbonate and sodium carbonate. Prior to DOC analysis, CO_2_ remaining in the sample water was removed by sparging with O_2_ gas. Then DOC concentrations were measured by high temperature combustion (680°C) on the same instrument.

DIC concentrations and alkalinity were used to determine pH and concentrations of field samples with the computer program CO2SYS [41], including contributions to alkalinity by SRP and Si and the effects of pressure (depth) ([42], refit by [43]). For the aquarium experiment, DIC concentrations were calculated using measured alkalinity and pH.

### Statistical analysis

The chemical and physical conditions above and underneath the algae (averaged over analytical triplicates/duplicates for each quadrat) were compared using paired t-tests. The effects of algal morphology and biomass on the ratios of above to underneath values of all parameters were tested with two-way analyses of variance. To assess differences in water quality and coral physiology in the laboratory experiment, two linear mixed effects models were used to test: (i) controls only (differences between illuminated and shaded controls, and between different times of sampling during the day), and (ii) shaded treatments only (differences between 0, 33, 67 and 100% algal water concentrations, and time of sampling). Maximum quantum yield data were arcsine square root transformed, while the RNA/DNA ratios were square root transformed to approximate normality. Data were analyzed with the statistical software package R (R Development Core Team [44]).
